# Collaborating with front-line healthcare professionals: the clinical and cost effectiveness of a theory based approach to the implementation of a national guideline

**DOI:** 10.1186/s12913-014-0648-4

**Published:** 2014-12-21

**Authors:** Natalie Taylor, Rebecca Lawton, Sally Moore, Joyce Craig, Beverley Slater, Alison Cracknell, John Wright, Mohammed A Mohammed

**Affiliations:** Centre for Resilience and Implementation Science, Australian Institute of Health Innovation, Macquarie University, Level 6, 75 Talavera Road, North Ryde, NSW 2109 UK; Institute of Psychological Sciences, University of Leeds, Leeds, LS2 9JT UK; Bradford Institute for Health Research, Bradford Royal Infirmary, Duckworth Lane, Bradford, BD9 6RJ UK; York Health Economics Consortium Limited, Level 2, Market Square, University of York, York, YO10 5NH England; Leeds Teaching Hospital NHS Trust, Beckett Street, Leeds, LS9 7TF UK; School of Healthcare Studies, University of Bradford, Bradford, West Yorkshire BD7 1DP UK

**Keywords:** Theoretical domains framework, Patient safety, Hospitals, Barriers, Interventions

## Abstract

**Background:**

Clinical guidelines are an integral part of healthcare. Whilst much progress has been made in ensuring that guidelines are well developed and disseminated, the gap between routine clinical practice and current guidelines often remains wide. A key reason for this gap is that implementation of guidelines typically requires a change in the behaviour of healthcare professionals – but the behaviour change component is often overlooked. We adopted the Theoretical Domains Framework Implementation (TDFI) approach for supporting behaviour change required for the uptake of a national patient safety guideline to reduce the risk of feeding through misplaced nasogastric tubes.

**Methods:**

The TDFI approach was used in a pre-post study in three NHS hospitals with a fourth acting as a control (with usual care and no TDFI). The target behavior identified for change was to increase the use of pH testing as the first line method for checking the position of a nasogastric tube. Repeat audits were undertaken in each hospital following intervention implementation. We used Zou’s modified Poisson regression approach with robust standard errors to estimate risk ratios for the use of pH testing. The projected return on investment (ROI) was also calculated.

**Results:**

Following intervention implementation, the use of pH first line increased significantly across intervention hospitals [risk ratio (95% CI) ranged from 3.1 (1.14 to8.43) *p* < .05, to 8.14 (3.06 to21.67) *p* < .001] compared to the control hospital, which remained unchanged [risk ratio (CI) = .77 (.47-1.26) *p* = .296]. The estimated savings and costs in the first year were £2.56 million and £1.41 respectively, giving an ROI of 82%, and this was projected to increase to 270% over five years.

**Conclusion:**

The TDFI approach improved the uptake of a patient safety guideline across three hospitals. The TDFI approach is clinically and cost effective in comparison to the usual practice.

**Electronic supplementary material:**

The online version of this article (doi:10.1186/s12913-014-0648-4) contains supplementary material, which is available to authorized users.

## Background

Clinical guidelines are an established and integral part of routine clinical practice [1]. Guidelines often summarise complex and rapidly changing research evidence with the aim of speeding up the translation of evidence into routine clinical practice and reducing unwarranted variations in the quality of care. More often than not, guidelines require health professionals to change their behaviour. Consequently, a range of methods have been developed to implement guidelines in routine practice – but, evaluations of these approaches have demonstrated variable effects [2,3]. This may be explained by the difficulties associated with changing behaviour, especially within complex social and environmental systems that demonstrate local variations [4]. As such, interventions to enhance the quality and safety of health care may be more effective when developed by those with local ‘expertise’ and tacit knowledge [5-7], and underpinned by evidence from behaviour change and implementation science literature [8-11].

One approach to implementation is based on a behaviour change methodology known as the Theoretical Domains Framework (TDF); e.g., [7,10,12]. The TDF aids the identification of barriers and levers to organisational and individual level behaviour change, which can be subsequently targeted with evidence based interventions. The framework comprises a description of the nature of the behaviour to be targeted and 11 domains of behaviour change (e.g., skills, beliefs about capabilities, social influences, etc.) which are based on theoretical constructs from multiple psychological and organisational behaviour change theories.

We developed a method for using the TDF during the implementation of clinical guidelines which draws on evidence based implementation principles, such as the need for management approval and on-going support [13], mapping of guidelines onto local problems [14], adopting the perspective of the target group [10], and co-design and production of interventions [13]. This Theoretical Domains Framework Implementation (TDFI) approach [15,16] involves a six step process for behaviour change: forming an implementation team; defining a locally relevant target behaviour; understanding barriers to performing the target behaviour; devising intervention strategies to address identified barriers; intervention implementation; and evaluation. Authenticating a bottom-up strategy, the TDFI approach uniquely aims to facilitate a collaborative team with a blend of front-line healthcare professional expertise and theoretical support to co-work through an implementation process.

Previous work has demonstrated the feasibility and acceptability of the TDFI approach for supporting the uptake of guidelines [15]. Therefore, the work reported here addressed the following research question*s*: 1) Can the TDFI approach improve the uptake of a patient safety guideline? 2) How clinically and cost effective is the TDFI approach for implementing a patient safety guideline in comparison to the usual implementation practice?

## Methods

### Setting

Medical Directors at 14 NHS hospitals from Yorkshire and the Humber region of the UK were contacted by the project team in April 2011 to inform them of the opportunity for their hospital to be involved in a project which aimed to support the implementation of national patient safety guidelines. In total, four hospital Trusts expressed an interest, and at the initial scoping meeting for each organisation, Medical Directors and senior management staff were asked to prioritise one or two guidelines. Three hospitals (from here on in referred to as H1, H2, H3)^a^ chose to focus on a common guideline released by the National Patient Safety Agency (NPSA) in March 2011: ‘reducing the harm caused by misplaced nasogastric (NG) feeding tubes’.

Fine bore NG tubes are frequently used in the clinical setting. The delivery of enteral feed through NG tubes that have been inadvertently placed in the respiratory tract is not uncommon and can have serious consequences; between 2005 and 2011, there were 21 deaths and 79 cases of harm in the UK due to feeding into the lungs [17], 50% of which were caused by misinterpretation of X-ray. Although there is no completely reliable method for checking tube placement, the NG tubes guideline provides a total of 17 recommendations for management/clinical staff for preventing feeding into the lung, including: pH testing should be the first line method used to confirm the tube is in the stomach, and that X-ray is used only as a second line test when no aspirate from the stomach can be obtained or the pH indicator paper has failed to confirm the tube position.

### Implementation method

The project staff worked with teams from each hospital using the six step TDFI approach to support the implementation of the NG tubes guideline. Full details of how each step was completed are reported elsewhere [15]. In summary the TDFI approach used six steps: 1) Team selection: multidisciplinary implementation teams (ranging from between 4–10 team members including doctors, nurses, and allied health professionals who were approached based on their expertise in, or enthusiasm for, the area of the guideline) were formed in each Trust; 2) Audit of current practice: staff audited NG tubes practice using co-developed audit tools to identify a target behaviour for change: increasing the *‘use of pH as the first line method to check tube position’* was the target behaviour identified by all three hospitals; 3) Identification of barriers to change: the Influences on Patient Safety Behaviours Questionnaire IPSBQ; [16] was distributed to relevant healthcare professionals in each Trust in both a paper copy and online format to assess the barriers to performing the target behaviour according to the domains of the TDF; 4) development of solutions to overcome barriers: following analysis of the questionnaire data, focus groups were held at each hospital with multi-disciplinary groups of staff from relevant wards/departments to discuss views regarding the specific target behaviour, and to devise interventions to overcome any barriers faced to performing this behaviour using guidance from evidence based behaviour change literature; 5) Implementation of solutions: a report outlining the process, findings, and suggested interventions was produced for each project and submitted to senior management in each Trust. Following authorisation for implementation, strategies were implemented; 6) Evaluation: post-intervention audits were undertaken by the implementation teams to determine the impact of the intervention on the performance of the target behaviour by staff within each organisation.

### Implementation tools

To support the above implementation methods, three implementation tools were required: an audit tool to assess performance – co-developed with each implementation team, the validated IPSBQ to assess barriers to change according to domains of the TDF, and a focus group schedule to develop further understanding about TDF barriers and to generate tailored implementation strategy ideas using TDF domain-mapped behaviour change techniques [18]. Details of how each of these tools have been developed are available elsewhere [15,16].

### Evaluation

#### Pre-post intervention implementation audits

The pre- and post-implementation audit data collected in each hospital was used to assess differences in practice following implementation of the strategies. Patient notes were audited by members of the implementation teams (Table 1) to find evidence about the indication for the NG tube, the process of tube insertion, and how the position of the tube was initially verified and monitored on a continuous basis. The implementation team members involved in tool development, who were assigned to reviewing notes, had discussed the process of completing the tool at length during implementation team meetings, where any uncertainties or discrepancies were resolved. Where possible, the same auditors were used to collect the data at both time points. For new case note reviewers (e.g., after hospitals rotations), experienced team members provided training using a ‘buddy system’ with an example set of case notes to ensure standardisation of the case note review approach. Depending on the complexity of notes being assessed, a single case note review took between 5 and 30 minutes. An example of the audit tool can be found in Additional file 1.

**Table 1:** **Data collection and intervention timeframes for each hospital**

**Hospital**	**Auditors**	**Pre intervention audit timeframes**	**Implementation time frames**	**Post-intervention audit timeframes**
H1	3 x junior doctors; 1 x registrar	1^st^ Jan-31^st^ Mar 11	1^st^ Sep 11-8^th^ Feb 12	9^th^ Feb-9^th^ May 12
H2	1 x consultant, 2 x junior doctors, 2 x registrars, 1 x medical student	1^st^ Aug-31^st^ Nov11	1^st^ May-31^st^ Aug 12	8^th^ Sept-1^st^ Nov 12
H3	3 x junior doctors, 2 x registrars	1^st^ Feb-30^th^ Sept 11	1^st^ Jun-15^th^ Sept 12	17^th^ Sept-4^th^ Oct 12
H4 (control)	3 x junior doctors, 1 x audit manager, 2 x dietician	1^st^ Jan-30^th^ Nov 11	N/A (control)	1^st^ Feb-31^st^ Dec 12

To access patient notes, a request was made to the medical records department in each hospital for notes of patients who had received an NG tube. In the intervention hospitals, this was determined by the identification of a specific code within the medical record indicating a patient had received a tube. In the control hospital, the electronic system did not contain this information. Therefore the filing system within the dietician office was the only method available to reliably identify patients who had received an NG tube; case notes were then requested from medical records. Case notes were delivered to a single main secure office in each hospital so that implementation team members could access and audit the notes. In H2 and H3 at both time points, both prospective and retrospective case note reviews were undertaken because the implementation team members could more easily access notes for patients on the wards as opposed to waiting for medical records to select notes and deliver to a single office. Within each individual hospital, the methods used to identify patients for each guideline were the same for each time point. Timeframes for data collection (Table 1) were different between hospitals due to the time-point at which an organisation agreed to be involved in the project, and/or the rate at which patients were receiving an NG tube (e.g., in some hospitals there were considerably more patients receiving an NG tube per week than in others).

#### Control hospital audit

A fourth hospital (H4), with whom we were working to implement a different patient safety guideline using the same TDFI process, acted as a control. H4 received the NG tubes NPSA guideline at the same time as the other sites (March 2011) and was provided with the same instructions by their local health authority – i.e., demonstrate evidence of implementation within a specified timeframe. Therefore, a standard a-theoretical NHS approach to implementation was undertaken in H4 as opposed to the TDFI theory based intervention.

Case note data for NG tube patients was collected over timeframes which encapsulated data collection periods from all three intervention hospitals pre- and post-intervention implementation. The same audit tool, auditing instructions, and processes for retrospectively identifying NG tubes patients were applied in the control hospital.

### Analysis

#### Outcomes for target behaviour identification

Given the key target behaviour for change was identified as increasing the *‘use of pH as the first line method to check tube position’*, ratios and percentages were computed for the indicator: pH used first line method (primary outcome variable). The following indicator data was also collected to provide information about what other (less desirable) methods were being used as the first line method to check the position of the tube: 1) X-ray; 2) tube placed in radiology (secondary outcome variables). Data for an additional secondary outcome variable – whether the method that had been used to determine tube position had been documented – was also collected. These data were used as the baseline measures of behaviour.

#### Measuring pre-post intervention implementation change

We used Zou’s modified Poisson regression approach [19] with robust standard errors to estimate risk ratios for the use of pH testing. The response variable was ‘pH testing used first line’ (yes/no). The no category incorporated ‘use of X-ray first line’, ‘tube placed radiologically’, and ‘no documentation of first line method used’. The covariates in the model were the hospital term and an interaction term with a binary pre/post variable (0/1). The coefficients from the model represent risk ratios, which separately compare the “rate of change” in each intervention hospital with the “rate of change” in the control hospital. We undertook a sensitivity analysis assuming no change in the control hospital to determine the extent to which our findings were robust to the reduction (not statistically significant) in pH testing seen in the control hospital.

### Ethics

Ethical approval was sought from the Bradford Research Ethics Committee. In view of the lack of involvement of patients, use of routine data and quality improvement focus of the project, it was classified as a service evaluation. Case note reviews were undertaken by trained healthcare professionals as part of the implementation approach. Focus group interviews with staff were undertaken following receipt of written informed consent; staff were made aware that all data collected would be anonymous and remain confidential.

### Return on investment

This project is one of a portfolio of projects contributing to the objectives of the Yorkshire and Humber Academic Health Science Network, in particular translating research and into practice (AHSN). NHS England, which licenses the AHSN, asked it to provide case studies showing the potential rate of return on investment (ROI) on these. This project was such selected as a case study and thus its methodology adopts a ROI approach for the Yorkshire and Humberside region.

The return on investment (ROI) was estimated using the formula [20]:

$$ \begin{array}{l}\sum \mathrm{Total}\ \mathrm{discounted}\ \mathrm{benefits}\ \mathrm{minus}\ \mathrm{total}\ \mathrm{discounted}\ \mathrm{costs}\\ {}\sum \mathrm{Total}\ \mathrm{discounted}\ \mathrm{costs}\end{array} $$

Benefits and costs were estimated for the Yorkshire and Humberside region with a population of 5.3 million. The analysis was based on a total of 34 acute hospitals within the region. Estimated benefits arose from:

- Change in use of methods used to check the position of the tube, including assessment of the second line methods used (i.e., if pH was used first line but the outcome was ‘unable to obtain aspirate from stomach’, or ‘pH >5.5’, what actions were taken, e.g., a) an additional attempt was made to obtain aspirate following re-positioning of the patient as per guidelines, b) the patient was sent for an X-ray, etc.).
- Reduced errors in reading X-rays and adverse events subsequently avoided;
- Replacing current practice – this was assumed to be dissemination of relevant safety awareness messages via emails and team meetings.

The main additional cost was for the intervention itself (see Additional file 2), which included: development and delivery of training and e-learning resources, poster and screensaver design and implementation, development of care pathway documentation, organisation of an awareness day/week, and the time devoted to the project by implementation team members. Estimates of resource use were obtained from hospital staff familiar with the intervention and current practice in order to minimise use of assumptions. The two main sources of unit cost information are “Unit cost of health and social care” [21] for cost of staff time and NHS Schedules of Reference Costs for examination and disease specific costs [22].

## Results

### Clinical effectiveness

**Figure 1:**
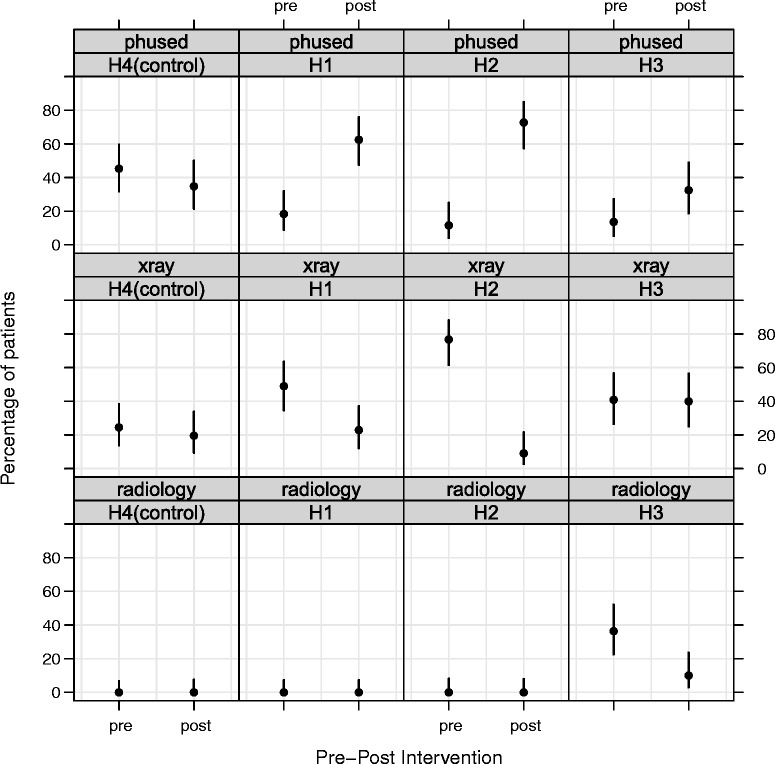
**Use of pH as the first line method for testing nasogastric tube position pre- and post- intervention implementation for H1-H4.**

Baseline data involving 43 to 53 patients and post-intervention data involving 40 to 46 patients are presented in Table 2 and graphically in Figure 1. The use of pH as a first line method at baseline was lower in the intervention hospitals (H1 = 18.4%, H2 = 11.6%, H3 = 13.6%) compared to the control hospital (H4 = 45.3%), whilst the use of X-rays was higher in the intervention hospitals (H1 = 49%, H2 = 76.7%, H3 = 40.9%) compared to the control hospital (H4 = 24.5%). The risk ratio for pH use in the baseline period for intervention hospitals ranged from 0.26 (95% CI = 0.11 to 0.62) to 0.41 (95% CI = 0.21 to 0.79) relative to the control (Table 3 and Additional file 3: Table S3.1)^b^. In the post intervention period, the use of pH testing increased significantly in the intervention hospitals (Table 2 and Figure 1). The relative risk of pH use after controlling for differences at baseline ranged from 3.1 (95% CI = 1.14 to-8.43, *p* < .05), to 8.14 (95% CI = 3.06 to 21.67, *p* < .001) compared to the control hospital, which remained unchanged (risk ratio (95% CI) = .77 (.47-1.26) *p* = .296).

**Table 2:** **Descriptive statistics for measurement indicators across each hospital**

**First line method**	**Hospital**	**Pre intervention (%)**	**Post intervention (%)**
pH test (↑)	H1	9/49 (18.4)	30/48 (62.5)
	H2	5/43 (11.6)	32/44 (72.7)
	H3	6/44 (13.6)	13/40 (32.5)
	H4 (control)	24/53 (45.3)	16/46 (33.3)
X-ray (↓)	H1	24/49 (49.0)	11/48 (23.0)
	H2	33/43 (76.7)	4/44 (9.1)
	H3	18/44 (40.9)	16/40 (40.0)
	H4 (control)	13/53 (24.5)	9/46 (19.6)
Placed in radiology (↓)	H1	0/49 (0)	0/48 (0)
	H2	1/43 (2.3)	0/44 (0)
	H3	16/44 (36.4)	4/40 (10.0)
	H4 (control)	0/53 (0)	0/46 (0)
Not documented (↓)	H1	16/49 (32.6)	7/48 (14.6)
	H2	4/43 (9.3)	8/44 (18.2)
	H3	4/44 (9.1)	7/40 (17.5)
	H4 (control)	16/53 (30.2)	21/46 (45.7)

(↑) & (↓) = desired direction of change.

**Table 3:** **Zou’s modified Poisson regression model coefficients estimating the risk ratios of the changes in the use of pH testing**

**First line method**	**Hospital**	**Pre intervention (%)**	**Post intervention (%)**	**Pre-intervention model coefficients with respect to the control hospital**	**p-value for model coefficients with respect to the control hospital**	**Post-intervention model coefficients with respect to the changes in risk in the control hospital**	**p-value for model coefficients with respect to the control hospital**
				**Risk ratio (95% CI)**		**Risk ratio (95% CI)**	
pH	H1	9/49 (18.4)	30/48 (62.5)	0.41 (0.21 to 0.79)	0.007	4.43 (1.99 to 9.87)	<0.001
	H2	5/43 (11.6)	32/44 (72.7)	0.26 (0.11 to 0.62)	0.002	8.14 (3.06 to 21.67)	<0.001
	H3	6/44 (13.6)	13/40 (32.5)	0.30 (0.14 to 0.67)	0.003	3.1 (1.14 to 8.43)	<0.05
	H4 (control)	24/53 (45.3)	16/46 (33.3)	Reference		0.77 (0.47 to 1.26)	0.296

For H1 and H2 the use of X-ray first line decreased (H1: from 49% to 23%; H2: from 76.7% to 9.1%), and for H3 the use of radiological insertion first line decreased between pre-post intervention implementation (from 36.4% to 10%). There was a 5% reduction in the use of X-ray first line in the control hospital (from 24.5% to 19.6%). H1 had an improvement in documented practice (from 32.6% missing documentation to 14.6%), whereas poorer documentation recording was found in H2 (from 9.3% missing documentation to 18.2%) and H3 (from 9.1% missing documentation to 17.5%) and the control hospital (from 30.2% missing documentation to 45.7%).

### Estimated cost effectiveness

Table 4 presents the pre-post intervention use and unit cost of diagnostic tests, with the main change being greater use of pH tests and fewer X-rays first line. Staff requirements to undertake the tests were advised by a nurse and radiographer at one site, whilst the cost of an X-ray was advised by a finance staff member. Applying the unit costs to the change in the usage of tests provides estimated savings across the Yorkshire and the Humber region of £0.51 million.

**Table 4:** **Usage of diagnostic tests pre and post intervention**

**Tests**	**Pre intervention**	**Post intervention**	**Unit cost per test**
	**First and second line %**	**First and second line %**	
pH test	14.1%	49.5%	£10.98
X-ray	60.2%	35.7%	£100
Placed in radiology	7.5%	1.4%	£128
Unknown	18.3%	13.4%	Assumed zero
Total	100.0%	100.0%	

The error rate in reading X-rays was taken from an audit of a similar intervention [23] and applied in this case. At baseline 192 X-rays were used to confirm placement of tubes, with 43 errors of interpretation found. Of these, seven placements were interpreted as unsafe when actually safe and five were in the lungs. After one year of implementing a project to improve the checking process, misinterpretation was found in one case across the three intervention hospitals. Avoided costs for each event include £43 for each tube re-inserted and £2,391 for the cost of pneumothorax injury [22]. The cost of a near-miss was estimated at £4,791, assuming a consultant-led team investigated the event (with staff members involved and duration of investigation advised by a clinician in H2). Total savings from fewer errors in reading X-rays were estimated at £60,427 per hospital, equivalent to £2.05 million across the region. Estimated savings of 10 minutes per year from no longer addressing the topic at team meetings were £14,970 per hospital, and £0.51 million for the region. Total estimated savings were £75,397 per hospital and £2.56 million across the region. The costs to deliver the intervention were estimated at £45,824 per hospital and £1.56 million across the region (see Additional file 3). Each hospital is assumed to hold an annual refresher at a cost per hospital of £18,192, equivalent to £0.62 million across the region. The estimated net savings per hospital in the first year are £29,573 rising to £57,205 in subsequent years and £1.00 m and £1.94 m respectively across the region.

The base case ROI, presented for 34 hospitals in the region, has a five-year time horizon, with costs and savings discounted at a rate of 3.5% per year. Sensitivity analyses are conducted assuming a 20% change in costs and savings. The ROI by year for the base case and various sensitivity analyses can be found in Additional file 4. The base case results show an excellent return even in year 1; the return improves each year with the improvement in diagnostic practice being maintained over time, but costs are lower because only a refresher course would be delivered in later years. The result is sensitive to both costs and savings.

## Discussion

The challenges associated with effective implementation of clinical guidelines are well documented [2,24-26]. These challenges are exacerbated with the ever expanding number of national guidelines (since 2001 the National Institute for Health and Care Excellence have published almost 200 clinical guidelines) [27]. Organisational approaches to dealing with this increasing volume of guidance tend to rely on top-down cascades of dissemination to clinical teams and self-reported compliance statements for assurance. This research aimed to discover whether an alternative, bottom up approach to implementation – working with frontline teams to identify and overcome key local barriers to performing a specific target behaviour – can improve the uptake of a patient safety guideline, and whether this is more effective than normal implementation practice.

Using the TDFI approach across three hospitals, we have demonstrated that co-designing interventions using evidence based strategies to address key barriers can significantly increase the uptake of national recommendations of NG tube positioning and significantly reduce the use of less safe behaviours. This is the first study to demonstrate how the TDFI approach is associated with change in specific behaviours to improve the uptake of a patient safety guideline across different organisations. This research highlights the importance of identifying key local barriers and co-designing interventions to elicit behaviour change.

When assessing the results for the three hospitals in more detail, it is evident that the trends for improvement in H1 and H2 are similar for the changes in the use of pH (H1 = 18.4-62.5%; H2 = 11.6-72.7%) and X-ray (H1 = 49-23%; H2 = 76.7-9.1%) as the first line method to check tube position. However, in H3, although there was an increase in the use of pH (13.6% to32.5%), which corresponded with a decrease in the number of tubes being placed in radiology (36.4% to 10%), results indicate that there were still a high proportion of patients being sent for an X-ray first line (40.9% to 40%) post-intervention implementation. Further discussions with staff would be needed in order to attempt to confirm those factors that contributed to this pattern of results and inform the refinements required to the implemented interventions. Nonetheless, these findings demonstrate that all the intervention hospitals improved in different ways, indicating the importance of an approach to guideline implementation that takes into account the local context. This may also offer one explanation of why top-down initiatives sometimes make little or no progress (as H4 showed).

The significant increase in the use of pH as the first line method for checking NG tube position has potentially prevented harm, especially in H1 and H2 where a corresponding reduction in the use of X-ray was demonstrated, the benefits of which are notable. For example, a decrease in the use of X-ray reduces the risk of X-ray misinterpretation – the biggest attributable cause of deaths to patients as a result of feeding into a misplaced NG tube [17]. Patients who have the position of their NG tube confirmed without the need for X-ray are also likely to be fed quicker – waiting times for an X-ray to confirm NG tube placement have been shown to take an average of four hours [23], and have less chance of encountering other sick patients as they are able to remain confined to their ward rather than being transported around the hospital. Furthermore, the return on investment analysis confirms potential savings are likely associated with a reduction in X-rays and adverse events avoided.

### Benefits and limitations of the TDFI approach

Benefits of the TDFI approach include the ability to identify different barriers across organisations associated with the implementation of the same guideline, demonstrating the validity of the approach. These differences in key barriers indicate the need for tailoring of interventions to local contexts. This is another facet that the TDFI approach can offer using barrier-matched theoretically underpinned behaviour change techniques [9] alongside principles of implementation, such as incorporating co-designed interventions into established structures [13,28,29]. At the same time, where there is some overlap between organisations for the presence of specific barriers, this allows for sharing of interventions and avoids unnecessary duplication. This occurred on an informal basis in the current project, and implementation team members indicated this was useful because it saved time and promoted inter-organisation working [15].

The limitations of using the TDFI approach itself include the complexities associated with working with healthcare professionals to identify a specific target behaviour for change. The TDF indicates that the barriers and levers affecting the performance of a single target behaviour should be identified. However, clinical guidelines frequently include a range of recommendations that may require behaviour change, so identifying the most important behaviour to focus upon can be challenging [16]. Additionally, the time, skills, and resources required by a nominated person to use this approach with teams to elicit behaviour change within a healthcare setting, in comparison to other methods of implementation, are as yet undefined. Investment in training those tasked with guideline implementation (e.g., quality managers) to use the TDFI approach, and undertaking longitudinal follow up with attendees to understand their experiences, and the extent to which behaviour change occurs, may be useful in this regard. Such activity would also fulfil the much needed translational gaps that currently exist by mobilising knowledge (i.e., clinical guidelines) into practice using an evidence based and pragmatic method.

### Strengths and limitations of evaluation

This is a pragmatic safety improvement project with an evaluation component. Evaluation of safety improvement initiatives is notoriously challenging [30], and pre-post intervention comparisons with a suitable control is frequently the most realistic approach [31]. Furthermore, this is the first time that ROI results have been presented, together with clinical outcomes from a TDFI project. To enhance ROI accuracy, resource use estimates were provided by clinical experts and unit costs come from national cost databases [22].

Despite our encouraging findings, there are methodological limitations of this study. Primarily, the hospitals were volunteers and were therefore not randomised to an intervention or control condition. The audit team were not blinded to case note reviews, and pragmatic factors meant that sometimes a mixture of retrospective and prospective reviews were undertaken, increasing the potential for bias. Furthermore, the control data we collected was from a single Trust on a retrospective basis, which encapsulated the timeframes covered in the pre- and post-intervention periods for which the data was collected in the intervention hospitals. This limits the extent to which we can be confident that the statistically significant differences we have found are reliable. However, these shortcomings are consistent with the well documented challenges associated with evaluating complex interventions in the healthcare setting, such as having to accept the restrictions that prevent the adoption of an ideal evaluation design, and the need to undertake evaluation alongside large scale implementation [32,33]. Nonetheless, the collection of additional data to provide a comparison to the intervention hospital data offers further support of the potential value of the TDFI approach and therefore warrants progression onto a more sophisticated research design. It would also be useful to test the effectiveness of the TDFI approach for the implementation of a range of guidelines to ensure it is not only generalizable across organisations, but also across different areas of healthcare professional behaviour change. Finally, although we have previously reported implementation team member perceptions of the feasibility, acceptability, and sustainability of this approach through post-implementation interviews [15], this was not undertaken as part of a formal process evaluation. A qualitative process evaluation e.g., [34,35] would have been valuable for providing some clarity around specific intervention effects.

The ROI approach also has limitations, including failing to capture other benefits particularly improved quality of life and the potential to reduce mortality. Moreover, some hospitals may elect to deliver the intervention and conduct refresher courses using different numbers and staff functions, and may not achieve and sustain the change in clinical outcomes observed in this study.

### Implications for practice and research

This work has revealed promise in the use of a theoretically informed framework for bottom-up implementation of guidelines, which provides a practical alternative to top-down implementation. It would be useful to further understand more about the transferability of the TDFI approach by assessing whether those tasked with eliciting behaviour change to improve the uptake of guidelines in healthcare organisations are able to apply the model in practice.

Implications for research include the need to test the TDFI approach under more robust research design conditions, to investigate the sustainability of the results (i.e., whether behaviour changes are maintained), and the generalizability of the methods for the implementation of different guidelines and across a range of settings. A crucial next step in the progress of this research will be to establish whether using a theoretically underpinned framework of implementation is more effective than providing support using implementation principles alone (e.g., adopting the perspective of the target group, obtaining management approval, mapping guidelines onto local problems). Furthermore, it would be useful to identify the extent to which the specific components of the approach (e.g., the identification of barriers and levers, co-designing interventions, using evidence based behaviour change techniques, etc.) contribute to success. Increasing understanding of exactly what works will enable evidence based refinements to be made to improve both the clinical and cost-effectiveness of this approach.

## Conclusion

We have demonstrated that the TDFI approach can improve the uptake of a national patient safety guideline across three healthcare hospitals, and is clinically and cost effective for implementing a patient safety guideline in comparison to normal implementation practice. The changes we have seen in healthcare professional behaviour as a result of this approach have the potential to reduce harm to patients and save lives.

## Endnotes

^a^H2 consisted of two hospitals representing one Trust, both of which were simultaneously involved in the project.

^b^(Additional file 2: Table S3.1) presents the results of the sensitivity analysis whereby Zou’s modified Poisson regression model coefficients estimate the risk ratios of the use of pH testing assuming no change in the control hospital. The control hospital results were adjusted to ensure there is no change from pre to post intervention. The results remain in the same direction but Hospital 3 no longer significant at 5% but is significant at 10%.

## Additional files

Additional file 1:
**Nasogastric tube audit tool example.** Contents of audit tool used to assess notes of patients who were fed through a nasogastric tube. Additional file 2:
**Intervention costs.** Intervention costs. Breakdown of costs for interventions. Additional file 3: Table S3.1. Zou regression sensitivity analysis. Zou’s modified Poisson regression model coefficients estimating the risk ratios of the use of pH testing assuming no change in the control hospital. Additional file 4:
**ROI base case and sensitivity analyses.** Return on investment base case and sensitivity analysis for year 1, and years 1&2, 1–3, 1–4, and 1–5.

## Abbreviations

TDF: Theoretical Domains Framework
TDFI approach: Theoretical domains framework implementation approach
ROI: Return on investment
NHS: National Health Service
NPSA: National Patient Safety Agency
NG: Nasogastric
IPSBQ: Influences on Patient Safety Behaviours Questionnaire
H1, H2, H3, H4: Hospital 1, Hospital 2, Hospital 3, Hospital 4
