# Supplementary material for: Collaborating with front-line healthcare professionals: the clinical and cost effectiveness of a theory based approach to the implementation of a national guideline
Source: BMC Health Serv Res. 2014 Dec 21;14:648. doi: 10.1186/s12913-014-0648-4 (PMC4301624; doi:10.1186/s12913-014-0648-4)
Supplement: Additional file 1: — Nasogastric tube audit tool example. Contents of audit tool used to assess notes of patients who were fed through a nasogastric tube. [file 12913_2014_648_MOESM1_ESM.pdf]

Additional File 1 – Nasogastric tube audit tool example

## Nasogastric tube audit

| | | |
|---|---|---|
| NHS number: ..................................................... | Ward Location: ............................................... | Date ..................................... |
| Patient age: ..................................................... | Name of staff carrying out assessment: ............................................................. | |

### SECTION 1: Insertion

1) What was the relevant diagnosis? (circle **one** as appropriate)

| | | | | |
|---|---|---|---|---|
| Stroke | Oropharangeal cancers | Motor disturbance | Decreased GCS | Other (specify): ............................................. |

2) What was the indication for the tube? (circle **one** as appropriate)

| | | | | |
|---|---|---|---|---|
| Feeding only | Feeding and medication | Medication | Decompression | Other (specify): ................................................. |

| | | |
|---|---|---|
| 3) Has the new care plan been used? | Yes | No |
| 4) Was the following information documented? | **Circle as appropriate** | |
| a) Risk assessment as per NPSA advice? | Yes | No |
| b) Nostril in which the tube was inserted into | Yes | No |
| c) Length of tube | Yes | No |
| d) Date and time (if provided, record time here: ..............................................) | Yes | No |
| e) Entry signed appropriately (i.e., in line with GMC guidance) | Yes | No |

5) Who inserted tube? (circle **one** as appropriate)

| | | | |
|---|---|---|---|
| Doctor (specify grade): ................................................ | Nurse (specify grade): ....................................... | Other (please specify): ................................................ | Not documented |

### SECTION 2: Confirming tube position

6) What was the **first line test method** to confirm position? (circle **one** as appropriate)

| | | | |
|---|---|---|---|
| pH paper | X-ray* | N/A (i.e., placed in radiology)* | Not documented |

*If pH paper method **was not used** as the first line test method, what was the reason given?
..........................................................................................................................................................................................

7) If pH paper method **was used**, what was the outcome? (circle **one** as appropriate)

| | | | |
|---|---|---|---|
| Successfully confirmed pH < 5.5 | Failed/unable to aspirate | pH indeterminate | Other (specify).................................... |

| | | | |
|---|---|---|---|
| 8) Was the patient sent for an X-ray? **(*if no, please go to Section 3: Maintenance of tube)** | Yes | *No | |
| 9) Was the purpose of the X-ray clearly documented on the request card? | Yes | No | ND |

10) Was the following documented? (circle **all** that are appropriate)

| | | | |
|---|---|---|---|
| X-ray result | Time and date of X-ray reviewed | Confirms correct patient and most current X-ray | Plan (i.e., safe to feed, etc.) |

| | | |
|---|---|---|
| 10) Was quality of the X-ray sufficient to determine tube location? **(*if 'No' please circle reason below)** | Yes | No* |

| | | |
|---|---|---|
| Over/underexposed | Patient rotated | Tip of tube missing off X-ray |

| | | |
|---|---|---|
| 11) Was the position of the tube correct? | Yes | No |

### SECTION 3: Maintenance of tube

| | | |
|---|---|---|
| 12) Was the NG tube position confirmed before each use (or at least once daily if continuous feeds)? | Yes | No |
| | Not documented | |
| 13) Please state the length of time patient was treated using NG tubes .................................................. | Not documented | |
| 14) How many times was the tube replaced? ................................................................................... | Not documented | |
| 15) Was care pathway/bedside documentation complete? (**Please state what was missing*): ......................................................................................................................................... | Yes | No* |
| 16) Any evidence that the patient suffered an adverse event as a result of the NG tube? ......................................................................................................................................... | Yes | No |
| 17) If patient now deceased, what was cause of death? ......................................................................................................................................... | | |

General comments about the notes you have seen (either positive/negative):
.........................................................................................................................................................................................
