# Supplementary material for: Collaborating with front-line healthcare professionals: the clinical and cost effectiveness of a theory based approach to the implementation of a national guideline
Source: BMC Health Serv Res. 2014 Dec 21;14:648. doi: 10.1186/s12913-014-0648-4 (PMC4301624; doi:10.1186/s12913-014-0648-4)
Supplement: Additional file 2: — Intervention costs. Intervention costs. Breakdown of costs for interventions. [file 12913_2014_648_MOESM2_ESM.pdf]

Additional File 2

Table 3.1. Zou's modified Poisson regression model coefficients estimating the risk ratios of the use of pH testing assuming no change in the control hospital

First Line method	Hospital	Pre intervention (%)	Post intervention (%)	Pre-intervention model coefficients with respect to the control hospital Risk Ratio (95%CI)	p-value for model coefficients with respect to the control hospital	Post-intervention model coefficients with respect to the changes in risk in the control hospital Risk Ratio (95%CI)	p-value for model coefficients with respect to the control hospital
pH	H1	9/49 (18.4)	30/48 (62.5)	0.41 (0.21 to 0.79)	0.007	3.38 (1.57 to 7.25)	0.002
	H2	5/43 (11.6)	32/44 (72.7)	0.26 (0.11 to 0.62)	0.002	6.20 (2.40 to 16.03)	< 0.001
	H3	6/44 (13.6)	13/40 (32.5)	0.30 (0.14 to 0.67)	0.003	2.36 (0.90 to 6.23)	0.082
	H4 (control)	24/53 (45.3)	21/46 (45.7)#	Reference		1	

#: Adjusted to ensure there is no change from pre to post in the control hospital

NB: Results remain in the same direction but Hospital 3 no longer significant at 5% but is significant at 10%.
