# Supplementary material for: Collaborating with front-line healthcare professionals: the clinical and cost effectiveness of a theory based approach to the implementation of a national guideline
Source: BMC Health Serv Res. 2014 Dec 21;14:648. doi: 10.1186/s12913-014-0648-4 (PMC4301624; doi:10.1186/s12913-014-0648-4)
Supplement: Additional file 3: Table S3.1. — Zou regression sensitivity analysis. Zou’s modified Poisson regression model coefficients estimating the risk ratios of the use of pH testing assuming no change in the control hospital. [file 12913_2014_648_MOESM3_ESM.pdf]

Additional File 3. Activities and cost per hospital

Activity	Cost	Driver
Audit	£7,266	100 people per audit pre and post, 15 minutes to retrieve data (band 5); process and present 4 weeks effort (band 7)
Materials	£237	DVD, poster and screen saver developed for region and each hospital shares costs
Senior management support	£1,127	5% of local team
Local team	£22,545	Three teams per hospital each comprising 1 radiographer; 1 consultant; 1 registrar; 1 nurse, 1 improvement lead. Each team devote 3 hours a week for 10 weeks
Project team	£1,179	One (band 7) Improvement Lead for 4 days
Time for training	£13,470	Training time 30 minutes per staff member: 621 staff per hospital receive this level of support.
Total	£45,824	
Total cost with 34 hospitals	£1,558,016	
