# Supplementary material for: Collaborating with front-line healthcare professionals: the clinical and cost effectiveness of a theory based approach to the implementation of a national guideline
Source: BMC Health Serv Res. 2014 Dec 21;14:648. doi: 10.1186/s12913-014-0648-4 (PMC4301624; doi:10.1186/s12913-014-0648-4)
Supplement: Additional file 4: — ROI base case and sensitivity analyses. Return on investment base case and sensitivity analysis for year 1, and years 1&2, 1–3, 1–4, and 1–5. [file 12913_2014_648_MOESM4_ESM.pdf]

Additional File 4. ROI base case and sensitivity analyses

Analyses	Year 1	Years 1 & 2	Years 1 to 3	Years 1 to 4	Years 1 to 5
Base case	82%	172%	204%	242%	269%
20% increase costs	52%	126%	153%	185%	208%
20% decrease costs	128%	240%	280%	328%	361%
20% increase savings	119%	226%	265%	310%	343%
20% decrease savings	46%	117%	143%	174%	195%
